# Analysis of a tobacco vector and its actions in china: the activities of japan tobacco

**DOI:** 10.1186/1617-9625-8-13

**Published:** 2010-10-27

**Authors:** Peisen He, Takeaki Takeuchi, Eiji Yano

**Affiliations:** 1 Department of Hygiene and Public Health, Teikyo University School of Medicine, Tokyo, Japan; 2 Department of Social Medicine, Harbin Medical University, Harbin, China

## Abstract

Japan Tobacco (JT) is the third largest tobacco company in the world, and China, the world's largest tobacco consumer, is one of the most important targets for JT. To provide information for tobacco control, we reviewed and analyzed JT and its tactics and strategies in the Chinese market mainly by systematic examination of documents which are made available in the University of California, San Francisco Legacy Tobacco Documents Library. As a result, JT has had a special interest to expand sales of its cigarettes in the Chinese market.

## Introduction

Smoking is a particular challenge for public health because unlike many other pathogens, this leading cause of morbidity and mortality worldwide continues to be actively promoted by large multinational corporations and governments. Previous writers have described the activities of the two largest transnational tobacco corporations (TTC) as summarized later. In this paper we report on the activities of Japan Tobacco (JT), the third largest TTC and one that, unlike Philip Morris (PM) and British American Tobacco (BAT), is largely owned and controlled by the Japanese government. We focus on JT's actions aimed at penetrating the Chinese market, the largest in the world and heavily dominated by another government tobacco monopoly, the China National Tobacco Company (CNTC).

The domestic tobacco business of JT in Japan faces an increasingly tough environment, as overall demand declines and competition with other tobacco companies intensifies. Growth in demand for cigarettes in Japan began to slow in the mid-1970s as a result of several factors, including an aging population, growing health concerns and price increases [1].

The proportion of smokers in Japan has been decreasing over the past 12 years. In 2007, smokers made up 26% of the adult population in Japan [2], down from 35.1% in 1996 and 32.9% in 2000 [3]. According to the smoking rate survey of Japan conducted in May 2008, 25.7% of Japanese adults smoke [4]. Besides, the 1987 suspension of import tariffs on cigarettes led to rapidly increased competition in JT's domestic tobacco market, which decreased JT's sales and market share. To combat the increased competitive pressures, JT has become more sophisticated and focused in their marketing efforts, transforming the company from a Japanese cigarette "manufacturer/distributor" to an International cigarette "manufacturer/marketer"[5,6]. One of its tobacco strategies is to "expand internationally into new markets to provide growth for the tobacco segment." Initially, "JT will target larger markets with a good opportunity, for gaining volume and share (e.g. China, Korea, Taiwan, Iran, etc.), and will invest in marketing and spending as required"[5]. Then in 1999, JT purchased the international tobacco operations of US multinational R. J. Reynolds and formed JTI, through which it operates its tobacco business outside of Japan [7].

Among JT's initial international markets, China is the largest tobacco producing and consuming country in the world, with a population of approximately 1.3 billion [8-10]. Meanwhile, China is facing a serious public health problem in active and secondhand smokers. Smoking is an important risk factor for chronic obstructive pulmonary disease (COPD), lung cancer and tuberculosis [11], which are the second, sixth and eighth leading causes of death in China, accounting for 2 million deaths in 2002 (20.5% of all death in China) [12]. China has over 350 million smokers and about 460 million secondhand smokers [8,13], and smoking contributed to about one million premature deaths annually [14]. If this pattern of smoking continues, premature deaths attributable to smoking can be expected to exceed two million deaths annually by 2020 [15]. In fact, JT knows "about one in seven non-smokers suffer respiratory illness because of exposure to smoke in the workplace" [16].

O'Sullivan and Chapman [17] and Lee et al [18] studied transnational tobacco company (TTC) industry documents and described TTC's aims and conduct in China, but JT was not mentioned because JTI was formed in 1999. Zhong and Yano [19] mainly described British American Tobacco (BAT)'s tactics and use of international trade policies to support their corporate interests. However, no previous papers have analyzed JT and examined their actions in China, except Assunta's doctoral thesis [6].

The objective of this study is to analyze JT and describe details of JT's strategies and tactics in China, in order to provide information for tobacco control in China and the world.

## Methods

For this study, we searched the on-line documents available from the University of California, San Francisco (UCSF) Legacy Tobacco Documents Library (LTDL) http://legacy.library.ucsf.edu and the relevant websites including Japan Tobacco (JT) http://www.jti.co.jp/JTI/, Japan Tobacco International (JTI) http://www.jti.com/, China National Tobacco Company (CNTC) http://www.tobacco.gov.cn/ and China's tobacco news website http://www.tobaccochina.com/.

The keywords we used include "China", "Japan Tobacco", "Chinese market", "China National Tobacco Company", and "State Tobacco Monopoly Administration".

In LTDL, the keywords were used in the basic and advanced search and we limited the time between 1998 and 2009. In the homepage of JT, JTI, CNTC and Tobacco China, we used the keywords in their search engine. We mainly analyze the documents that were related to JT itself and JT's marketing in China. A panel constitutes one researcher and one expert on tobacco study. The panel members reviewed the manuscripts that were searched through from the site mentioned above in meetings that were conducted twice a month for a total of 3 months. The Delphi method was used to select appropriate papers for JT information related to China [20]. Documents that were illegible, duplicated, about the tobacco technology and other documents judged to have little interest to our aim were excluded.

In LTDL, we have reviewed thousands of documents, but only a few documents have relevance to our study objective. Thus, compared to PMI and BAT, JT has released fewer documents and especially the documents related to China. One of the most important reasons is that JT was protected by the Japan Tobacco Inc. Law and Tobacco Business Law of 1984. Even the Japanese courts do not have the power to subpoena the company's internal records, which has made it difficult to document JT's strategies [21].

## Results

The LTDL search identified 1181 documents, of which 1142 did not meet one or more of the inclusion criteria, leaving 39 documents for inclusion in the present review. Besides, there were 12 relevant documents that were found from the websites of JT, JTI, CNTC and Tobacco China. As a result, 51 relevant articles were included for our analysis.

### China's tobacco market

China is by far the world's largest cigarette market, accounting for over 30% of total world consumption. The Chinese cigarette market is nearly three and a half times the size of the US market and over 12 times the size of the German market, the largest in Western Europe [10].

The Chinese population is over 1.3 billion constituting 23% of the world's population. In addition, in China, 63% of adult males and 4% of females smoke, with 75% of males starting before the age of 24 [22].

The Chinese cigarette market is dominated by a state-owned monopoly, the State Tobacco Monopoly Administration (STMA), which strongly protects the domestic tobacco industry [23]. The STMA controls all aspects of the tobacco industry including tobacco growing, processing, product manufacturing and distribution and also material and machinery supplies, all of which are operated through the China National Tobacco Corporation (CNTC) [10]. CNTC produces over 1.7 trillion cigarettes annually [24]. Even a small share of such a huge market would mesmerize any tobacco company.

### CNTC and other TTCs

Philip Morris International (PMI) is the leading international tobacco company, with products sold in approximately 160 countries [25]. PMI and CNTC signed an agreement for the production and marketing of "Marlboro" cigarettes, as well as other brands, in China beginning in 1994. The cigarettes have been produced in existing CNTC factories and have been for both domestic consumption and export. The agreement would not require any initial capital investment by PMI, but PMI must introduce technology, training and human resources expertise to the CNTC's factories [26]. On 21st December 2005, the CNTC and PMI announced the establishment of a long-term strategic cooperative partnership further. Agreements provided for the licensed manufacture and sale of "Marlboro" cigarettes in China and establishment of an international equity joint venture company in Switzerland. In accordance with relevant provisions of the Law of the People's Republic of China on Tobacco Monopoly, "Marlboro", owned by PMI, will be produced under license at CNTC's affiliate factories, and will be distributed by CNTC's official distributors nationwide in China. The international joint venture company to be established by China National Tobacco Import & Export Group Corporation (CNTIEGC) and PMI, in which each party will hold 50% of the shares of the company, will be based in Lausanne, Switzerland. Following its establishment, this joint venture company will offer consumers a comprehensive portfolio of Chinese heritage brands globally, expand the export of tobacco products and tobacco materials from China, and explore other business development opportunities. The joint venture company will utilize both CNTC's and PMI's extensive sales and distribution infrastructure, financial resources and management experiences to develop business opportunities worldwide [27].

British American Tobacco (BAT) is the world's second largest tobacco group by global market share, with brand sold in more than 180 markets [28]. BAT China is one of BAT's subsidiaries, and existed in China in 1903 already. At present, BAT China is the one-up international tobacco company in China and set up the agencies in many major cities. The products of BAT are imported and distributed by CNTC, and the main products in China are "SE555", "Kent" and "Hilton" which are popular with Chinese smokers [29].

BAT entered into China when it was established at first, and was forced to leave China in 1956. In the 1980s, BAT came back to China again, and cooperated with Wuhu Cigarette Company to produce cigarettes named "Dubao" at that time. From then on, BAT cooperated with CNTC to manufacture a series of joint venture brand, including "Coco", "Huang Guo Trees", "Le Fu Door" and so on. In the tobacco leaf field, BAT and CNTC has been cooperating with each other for almost 10 years [30]. In the middle of the 1990s, BAT began to "help China's agriculture" by setting up tobacco leaf base and guiding the tobacco farmers to grow the tobacco leaf [30]. Besides, BAT China has established the cooperation plan on the tobacco leaf growth in Yunnan and Sichuan province of China [29].

Although CNTC has closer relationship with foreign tobacco companies year by year, the STMA still keeps foreign manufactures on tight rein. Importation allotment, marketing and distribution are under direct supervision of the CNTC. Legal and genuine foreign cigarettes make up only an estimated 4 percent of China's market. All marketing activities are handled through the STMA's China National Tobacco Sales & Marketing Corporation. Marketing opportunities are, however, limited. Asla, a spokesperson for Philip Morris, says, "Print, radio and television advertising are banned nationwide. Outdoor advertising is permitted only with prior approval, and is banned in over 70 local jurisdictions." To get the word out in big cities, foreign manufactures often sponsor events and parties at nightclubs. A common sight on Beijing's San Li Tun Bar Street, a street of night spots for Beijing's middle and upper class, are the "cigarettes girls." Attractive young women dressed in Cigarettes Company logos pass out free samples, lighters and other promotional items to patrons [31].

The STMA is out to protect its own. Even with WTO accession, STMA will remain the dominating force of China's tobacco industry [31].

### Japan Tobacco Company Profile and Its Market Share in China

JT is a joint stock corporation that was incorporated in April 1985 under the Commercial Code of Japan, pursuant to the JT Law [1]. The history of JT dates back to 1898, when the government formed a monopoly bureau to operate the exclusive sale of domestic leaf tobacco. In the early 1900s, the government extended this monopoly to all tobacco products in Japan and to the domestic salt business. In 1949, the bureau became the Japan Tobacco and Salt Public Corporation (JTS), which was assigned the role of the country's sole producer and supplier of tobacco and sole purchaser and supplier of salt products [5].

Between 1981 and 1985, following the demand of US tobacco industries, US government pressured Japan to reduce tariff for tobacco import and free access to the Japanese market [32]. In 1985, the company was changed from one of governmental sectors to a private company under the name Japan Tobacco Inc., though 100% of its share was owned by the government. At the same time, the Japanese market was fully opened to foreign tobacco products. In April 1987, all import duties were removed from foreign cigarettes under the strong pressure from the USA [21] who wanted to ease the trade imbalance between USA and Japan. This brought down the price of foreign cigarettes and increased the market share of imported cigarettes dramatically [5]. JT was semi-privatized in the same year, and two thirds of the company was owned by Japan's Ministry of Finance [21]. Beginning in 1988, JT began focusing efforts into diversification with the establishment of JT Agris Corporation in agribusiness, JT Engineering Inc. in systems engineering and Lifix Inc. to increase participation in the pharmaceutical industry [5]. According to JT's management vision developed in 1995, JT proposed to concentrate in three strategic areas: (1) foreign business; (2) medical business and (3) food [33].

Nowadays, JT is striving to become a "global growth company that develops diversified, value-creating businesses" [1]. JT's domestic tobacco market was in the charge of JT's headquarters, and the domestic market share is 65.1% in the fiscal year of 2009 [1]. In the domestic tobacco market, total demand for cigarettes has continued to decline and increases in the tobacco excise tax that took effect in July 2003 and July 2006 had an additional impact on the market. Meanwhile, as tobacco-related regulations are being strengthened in various ways, market share competition with foreign brands is intensifying. JT hopes to overcome these difficulties, in order to ensure that the domestic tobacco business continues to serve as the JT Group's core source of profits [1].

**Figure 1:**
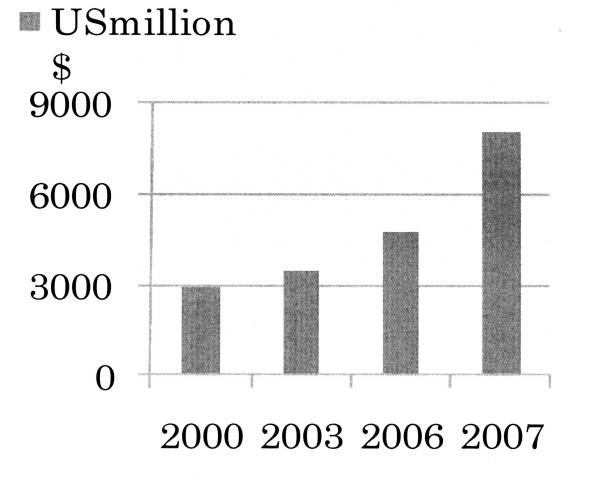
**The Increase of JT's International Sales after Taxes**. **Source**: reference [55].

The world market share of JTI is 10.6% and the products of JTI were sold in over 120 countries in the fiscal year of 2008 [1]. In 2007, Gallaher Group Plc was acquired by JT, which leads to sharp increase of international sales (Figure 1). Now, JTI's business covers almost all over the world, including North & Central Europe (UK, Ireland, Sweden, and Austria), Commonwealth of Independent States, namely CIS+ (Russia, Kazakhstan, Ukraine and Romania), South & West Europe (such as Spain, France, Italy and Greece) and Rest of the world (Taiwan, Malaysia, Turkey, Canada etc.). Based on the Global Flagship Brands (GFB) containing Winston, Camel, Mild Seven, Benson & Hedges, Silk Cut, LD, Sobranie and Glamour, international tobacco business has been continuing to be the profit growth engine of the JT group [1].

JT has been concentrating its sales in China for a long time because "the Chinese Market is extremely large, and is estimated to be worth an annual 1.7 trillion dollars. The market is expected to continue to grow and JT plans to continue strengthening its sales strategies in China" [34]. Between 1989 and 1992, JT's cigarettes accounted for 1.1%-1.5% among the all tobacco products imported from foreign countries by China [5]. In 1995, China's imports from Japan (which represent mainly JT's Mild Seven brand) were about 0.43 billion pieces, which accounted for about 0.025% in Chinese cigarette markets then [10]. In 2001, the market share of JT in China was about 0.1% [35]. However, fortunately total tobacco sales in the mainland of China have not increased as of 2009 [1].

### Advantages of JT

JT has some unique advantages relative to other TTCs, such as limited litigation history, no transparency about its activities because it was not included in the Master Settlement Agreement and government support.

### Little litigation

Compared to PMI and BAT, JT has little litigation. There are only 24 litigation cases related to JT by the end of May 2008, while, there are 137 and 3329 aiming to PMI and BAT respectively by the end of December 2007 [36]. JT and its subsidiaries have never lost a case or paid any money to settle a case out of court [1].

### Not in the Master Settlement Agreement

In 1998, the availability of tobacco industry documents increased exponentially as a result of the settlement of a suit by the state of Minnesota and Blue Cross/Blue Shield against the major tobacco companies. As part of the Minnesota settlement, Attorney General Humphrey required that the defendants release millions of pages of internal documents. These documents are stored in depositories in Minnesota and Guildford, England. The Master Settlement Agreement between the attorneys general of 46 states and B&W/BAT, Lorillard, Philip Morris, RJ Reynolds, the Council for Tobacco Research, and the Tobacco Institute required that the documents be made available on the Internet and be updated until 2010 [37]. JT is not in this agreement.

### Supported by the Japanese government

In 1985, JT was established under the Japan Tobacco, Inc. Law ("the JT Law") for the purpose of developing businesses related to the manufacture, sale, and importation of tobacco products. In advance, the Tobacco Business Law was enacted in August 1984 for the purpose of achieving sound growth for Japan's tobacco industry, securing stable government revenues, and contributing to the healthy expansion of the Japanese economy [1]. Because of this law, Japan can not declare reduction of smoking in the below mentioned "Health Japan 21st" project despite its ratification of the WHO Framework Convention on Tobacco Control (FCTC).

Japan ratified the FCTC in June 2004, being among the first 20 countries to do so [38]. Among the negotiations for the FCTC, Japan's participation presented particular interest of tobacco industry. At the first two Intergovernmental Negotiating Body sessions (INB), the Japanese delegation consisted mainly of representatives of the Ministry of Health, Labor and Welfare (MHLW) and had no representation from the Ministry of Finance (MOF). However, the Japanese delegation consisted of 20 members at the 6th Intergovernmental Negotiating Body session, by which time there were as many representatives from the MOF as there were from the MHLW [39]. The MOF has direct and indirect influence over JT, including its overall policies. The participation of the ministry may be seen as representing tobacco industry interest [40]. Similarly, Japan's Ministry of Agriculture, Forestry and Fisheries has historically supported tobacco interests because of Japan's sizable tobacco agriculture crop and the strong political constituency of tobacco farmers [41]. At last, the Japanese delegation successfully weakened the FCTC by arguing for extensive optional language [39], which is good for the Japanese tobacco industry--JT.

In Japan, there is a 10 year nation-wide health program---"Health Japan 21st", which was approved by the cabinet. In the Health Japan 21st, there are many numerical goals including regular exercise, suicide reduction and prevention of heavy drinking, but a target for smoking cessation was not included. Before 2000, Japan's MHLW once stated a goal of halving the prevalence of smoking by 2010 [42]. However, after strong opposition by tobacco industry, Ministry of Agriculture, and the MOF, even the reduction of adult smoking was not mentioned. Only remained were prohibition of under age smoking, support smoking cessation for those who want to quit smoking, and dissemination of knowledge for health hazard caused by tobacco.

Besides, many of the company executives of JT were retired officers of MOF. For the first 15 years after JT's privatization in 1985, the top position of JT has been regarded as the next job for the top officers of MOF. All of the three former CEOs of JT had been either ex-Deputy Minister of MOF or ex-Director General of the National Tax Agency [43].

### The cooperation between JT and China

JT started to cooperate with China in the 1980s. In order to compensate for the reduction in the volume of sales of cigarettes on the home market and for export, the Japan Tobacco International Corporation (JATICO, later JTI) was established in April 1984. In the middle of 1984 JTS and CNTC reached an agreement on the arrangement for an exchange of technology. Afterwards, JT opened an office in Beijing of China in February 1985. Meanwhile China had imported cigarettes from Japan at a value of five million dollars while Japan had purchased from China over 1000 tones of leaf tobacco and fairly large quantities of Salt. The main JT's products in China at that time are Mild Seven, Seven Stars, Hope, Peace, Castes, Hi-Lite [44]. JT's important international market contains Taiwan, Korea, and mainland of China at the earlier time [44].

Besides, JT has been cooperating with CNTC to produce "Youqi" and "Huaying", and some "Camel" cigarettes are produced in Yanji Cigarettes Factory in Jilin [45]. In fact, Yanji Cigarettes Factory in Jilin cooperated with RJ Reynolds at first. RJ Reynolds had concluded a licensing arrangement with Yanji Cigarettes Factory for the local production of "Camel", which is being announced in December 1998. In March 1999, JT took control of RJ Reynolds' international operations, and consequently since then, JT cooperates with Yanji Cigarettes Factory rather than RJ Reynolds [10].

JT's former President Masaru Mizuno once said in an interview "JT will continue looking for alliance with overseas companies so that it can survive a drop in domestic demand for cigarettes. China could be a big market in the future, though it depends on the way China joins the WTO. We have jointly developed a new brand of cigarettes with a Chinese company, and soon that company will start manufacturing their products" [46]. The company Mizuno referred is Shanghai Gaoyang International Tobacco Co. JT began to make cigarettes in China from 2000 for marketing its own brands. JT hoped to tap the Chinese market and planned to turn out 400 million cigarettes a year in Shanghai, marketing them under its top-selling brands such as Mild Seven and Mild Seven Light. This was its first local production in China. After that JT looked into cooperating with the Shanghai firm in low-tar, low-nicotine production technology and product development for the Chinese market [47].

In addition, JT also developed the market for Shanghai Tobacco Company in Japan through its own channels. In November 2003, "Zhong Hua", "Double Happiness" and "Golden Deer" of Shanghai Tobacco Corporation entered Japan's market by Unitobacco which is JT's subsidiary in charge of distributing imported cigarettes [45]. Technically speaking, Shanghai Tobacco Corporation entering into Japan means the loss of JT's domestic market share. What JT did is in order to keep a good relationship with CNTC and increase their market share in China in the future [45].

### JT's marketing activities in China

Before China joined the WTO, JT already knew "our sales may not expand rapidly in China in the near future because of high tariffs and distribution network restrictions. But it is a promising market because of its size and recent economic progress." Thus, JT has maintained 11 offices in China before 2000, which are preparing for brisk business in the future [48]. In addition, the CEO of JT said "JT will not lose Chinese market by no means once China opens its market." "We must pay special attention to Chinese market, especially its international commercial conditions. It is not an easy thing to enter into China....... But I think our products will have some perspective in Chinese market according to our sales situation in Korea and Taiwan." [45]

In January 2003, JT established the China Division especially for Chinese market under its Tobacco Headquarters. The division centralizes two functions: the formation of key relationships in the Chinese market through technical support and other activities, which had previously been handled by the JT parent company, and the marketing of tobacco products, which had been handled by subsidiary JTI. The aim of this reorganization is to strengthen its medium- and long-term activities in the Chinese market in view of China's entry into the WTO [49].

In recent years, JT has set up clubs named "Mild Seven Times" in some big Chinese cities, the service of which provide has no relevance with JT's cigarettes brand on the surface. Though in reality, "Mild Seven Times" has the function of popularizing JT's brand among Chinese smokers, so JT's cigarettes are quietly advertised in Chinese market in a "peaceful and friendly" way. JT also sponsored the "Mild Seven Outdoor Quest" in order to do advertisement for its brand in China, which is a four-day stage adventure race, covering approximately 300 kilometers and containing 7 events: mountain biking, kayaking, in-line skating, adventure skills, paddling and running. "Mild Seven Outdoor Quest" has great effect on the targeted consumers, especially the young people [45]. JT is only allowed to sponsor these events in China and Malaysia because of stringent laws, so lack of local regulation in China and Malaysia provide an opportunity for JT to do this. Mild Seven Outdoor Quest was famous for triathletes, it was held in China from 1997 to 2001 and in Malaysia from 2002 to 2004, the race was finished in 2005 [50].

## Discussions

### JT's ambition in China and the issues of public health

Although it is not publicly recognized, JT is very active in China today. Even if China has begun to restrict tobacco advertising, promotion and sponsorship on radio, television, printed media and the internet [51], JT still adopts other strategies to advertise its products in China. For example, it has established "Mild Seven Times" in some cities, sponsors "Mild Seven Outdoor Quest", and particularly "China Division" was established in JT specified for Chinese market. All of these actions revealed JT's ambition in China. The fact that Chinese business was transferred from JTI to China Division in JT headquarter demonstrates that JT has paid more and special attention to Chinese market. In JT's Annual Report 2004, "Net sales from the domestic tobacco business increased despite the drop in the sales volume of tobacco products in Japan." Because "the impact of decline in sales volume was offset by the inclusion of net sales in China, Hong Kong and Macau as part of the domestic tobacco business starting in the fiscal year ended March 31, 2004" [52]. So it's obviously that JT has treated Chinese tobacco market as its domestic market, which discloses JT's ambition in China.

In addition, cigarette smoking causes an enormous economic burden in China through a huge number of preventable diseases, health care costs, premature deaths, and productivity losses. The total economic costs had grown from $3.3 billion in 1989 to $5.0 billion in 2000 [53]. Since the health effects of smoking on morbidity and mortality are cumulative, China will bear a much heavier economic burden from cigarette smoking in the future if the current trends in smoking behavior continue [54]. Because more than half of the stock of JT is held by the Japanese government, the Japanese people may be forced to bear the huge burden of litigation and claim for compensation in future. So far, JT suffers from burden of litigations very little but the situation may change at any moment coming from any place due to its worldwide and rapidly expanding business.

Form the angle of all over the world, Tobacco has already killed 100 million people in the 20th century, and 5.4 million deaths per year currently result from tobacco use. In the absence of urgent action, more than 8 million tobacco-related deaths are predicted for every year beginning in 2030, and approximately 1 billion tobacco-related deaths are predicted to occur during the 21st century [54]. We must intensify attention to the role of tobacco companies as vectors of smoking and emphasize responsibilities of the Japanese government for tobacco control.

### Limitations

We admit that the search for documents is incomplete. Firstly, the valuable documents released by JT are so few. Secondly, we only mainly choose the documents which are between 1999 and 2009. Thirdly, the number of documents in LTDL and other related website is continually expanding as time goes on, so we undoubtedly do not analyze the documents after our given period.

## Conclusions

JT has adopted lots of novel and effective strategies in China which demonstrates that JT has great ambition in the Chinese market. JT's activities are threat to humans' health and the movement of tobacco control. We must focus more on drawing attention to the responsibility of tobacco companies for tobacco control.

## Competing interests

The authors declare that they have no competing interests.

## Authors' contributions

PH carried out study design, study protocol, sample collection, references collection and manuscript drafting. YE and TT took part in revision of manuscript. All authors read and approved the final manuscript.
